# Obesity and neuroinflammatory phenotype in mice lacking endothelial megalin

**DOI:** 10.1186/s12974-017-0800-2

**Published:** 2017-01-31

**Authors:** Fernando Bartolome, Desiree Antequera, Eva Tavares, Consuelo Pascual, Rosario Maldonado, Antoni Camins, Eva Carro

**Affiliations:** 1 Neurodegenerative Disorders Group, Instituto de Investigacion Hospital 12 de Octubre (i + 12), Madrid, Spain; 2 Centro de Investigación Biomédica en Red de Enfermedades Neurodegenerativas, CIBERNED, Madrid, Spain; 3 Clinical and Experimental Pharmacology Research Unit, Valme University Hospital, Seville, Spain; 4 Unitat de Farmacologia i Farmacognòsia, Facultat de Farmàcia, Institut de Biomedicina de la UB (IBUB), Universitat de Barcelona, Barcelona, Spain

**Keywords:** Megalin, Obesity, Leptin resistance, Hyperleptinemia, Blood–brain barrier, Inflammation

## Abstract

**Background:**

The multiligand receptor megalin controls the brain uptake of a number of ligands, including insulin and leptin. Despite the role of megalin in the transport of these metabolically relevant hormones, the role of megalin at the blood–brain-barrier (BBB) has not yet been explored in the context of metabolic regulation.

**Methods:**

Here we investigate the role of brain endothelial megalin in energy metabolism and leptin signaling using an endothelial cell-specific megalin deficient (EMD) mouse model.

**Results:**

We found megalin is important to protect mice from developing obesity and metabolic syndrome when mice are fed a normal chow diet. EMD mice developed neuroinflammation, by triggering several pro-inflammatory cytokines, displayed reduced neurogenesis and mitochondrial deregulation.

**Conclusions:**

These results implicate brain endothelial megalin expression in obesity-related metabolic changes through the leptin signaling pathway proposing a potential link between obesity and neurodegeneration.

**Electronic supplementary material:**

The online version of this article (doi:10.1186/s12974-017-0800-2) contains supplementary material, which is available to authorized users.

## Background

Megalin, also called low-density lipoprotein receptor-related protein-2 (LRP2) is a multiligand endocytic receptor that belongs to the LRP family proteins [1]. Megalin is expressed at the blood brain barrier (BBB), where it binds and internalizes a wide variety of ligands, including hormones, such as leptin [2, 3] and insulin [4]. Interestingly, megalin binds amyloid-beta (Aβ) peptide contributing to brain Aβ clearance, one of the histopathological markers of Alzheimer’s disease (AD) hence low megalin levels are related to AD pathology [5, 6].

Leptin is a canonical weight-regulatory hormone that modulates body weight by reducing food intake and increasing energy expenditure [7]. Its action is mediated by interaction with the leptin receptor (ObR) that is mainly expressed in the hypothalamus but also in other cerebral regions, including cerebral cortex and hippocampus [8]. To reach these target areas, leptin binds to the ObR once it passes through the BBB [9]. Thus, leptin delivery into the central nervous system (CNS) seems to represent a crucial step toward the regulation of food intake and energy balance. We previously generated a transgenic mouse model with lack of megalin in the brain endothelial cells (EMD mice) [10]. We found that EMD mice exhibited cortical neurodegeneration and cognitive deficits, including increased anxiety and impaired learning ability and recognition memory. These results suggested that EMD mice could reproduce several clinical features described in neurodegenerative disorders.

In this study, we investigated the brain megalin-mediated leptin signaling in the EMD mice. We found that EMD mice exhibit mild obesity as a result of hyperphagia, increased adiposity, elevated leptin and insulin plasma concentration, hypertriglyceridemia, impaired glucose tolerance, and decreased hypothalamic leptin signaling. Moreover, EMD mice lead to neuroninflammation, by triggering several pro-inflammatory cytokines, and impaired neurogenesis. In summary, deletion of megalin in brain endothelial cells was found sufficient and necessary to promote obesity and activate obesity-induced neuropathological mechanisms, including neuroinflammatory processes.

## Methods

### Animals

A number of 12 male EMD mice were generated using the Cre-Lox system under the control of the Tie2 as previously reported [10]. A number of 10 nontransgenic littermates (i.e., Tie2-Cre^−^mice) were used as controls (megalin ^flox*/*flox^) and named as control mice. EMD and control mice were grouped under a 12 h light: 12-h dark schedule and allowed ad libitum access to food and water. Body weight was monitored weekly throughout the study. Daily food intake was calculated as the average intake of chow. At the end of the experiments, animals were anesthetized with isoforane, blood was drawn and perfused transcardially with saline buffer or 4% paraformaldehyde in 0.1-M phosphate buffer (PB, pH 7.4) for biochemical and immunohistochemical analysis, respectively. Then, brain, liver, and adipose tissue were collected for further processing and stored at −80 °C until analysis. The liver and adipose tissue were previously weighed. Glucose tolerance test was assessed prior to sacrifice. All animals were handled and cared for according to the Council Directive 2010/63/UE of 22 September 2010.

### Analytical procedures

Plasma concentrations of leptin and insulin were measured by ELISA using mouse standards according to the manufacturer’s guidelines (mouse leptin ELISA kit; mouse rat insulin ELISA kit, from R&D). Plasma triglycerides (TG) were quantified using the commercially available kits (Roche Molecular Biochemicals). Glucose tolerance test was performed in 16-h-fasted animals. After collection of a baseline sample, mice received an intraperitoneal (i.p.) injection of glucose (2 g/kg body weight). Blood glucose levels were detected after 15, 30, 60, and 120 min after glucose injection, using a Compact Plus Glucose Meter (Accu-Chek, Roche). Area under the curve (AUC) was used to compare glucose tolerance between groups.

### Immunohistochemistry

For immunohistochemistry assays, fixed brains were cut on a vibratome (Leica Microsystems) at 30 μm, and tissue sections were collected in cold 0.1 M PB, After overnight incubation, primary antibody staining was revealed using fluorescence-conjugated secondary antibodies from Molecular Probes. Primary antibodies were: rabbit anti-NeuN (Millipore), mouse anti-leptin receptor (Obr, Santa Cruz Biotechnology), mouse anti-GFAP (Sigma-Aldrich), mouse anti-phosphorylated STAT3 (pSTAT3, Cell Signaling), rabbit anti-ionized calcium-binding adaptor molecule 1 (Iba1, Wako), goat anti-doublecortin (DCX, Santa Cruz Biotechnology), and mouse anti-polysialylated neural cell adhesion molecule (PSA-NCAM, Chemicon). Fluorescent images were obtained by laser confocal microscopy (Carl Zeiss-LSM510) using the excitation lasers 405, 488, and 568 for DAPI, green, and red fluorescence, respectively. For quantitative analysis of hypothalamic Obr^+^/NeuN^+^ neurons and Obr^+^/GFAP^+^ astrocytes, double fluorescence cells were counted, marked to prevent multiple counts, and expressed as percentage of total GFAP^+^ or NeuN^+^ double-labeled cells. For quantitative analysis of hypothalamic pSTAT3^+^/NeuN^+^ neurons, double fluorescence cells were counted, marked to prevent multiple counts, and expressed as percentage of total NeuN^+^ neurons. The number of reactive microglial Iba1^+^ cells was estimated by unbiased stereology method as previously described [11]. GFAP^+^ area was stereologically analyzed as described previously [12]. In addition, one series of sections was used for double-labeling experiments using DAPI nuclear staining (Sigma) and DCX as a marker of neuronal progenitor cells. Using the Cell Counter program of the NIH ImageJ software, we counted hippocampal DCX^+^ cells of the captured confocal microphotographs.

### Mitochondrial mass assessment

Control and EMD cortical sections were incubated with the mouse primary antibody anti-ATP synthase β subunit (Abcam, Cambridge, UK) targeting mitochondria. 4–5 high-resolution Z-stacks were acquired corresponding to different regions from cortical tissue sections per animal from each group (control = 10 and EMD = 12). The method enabled adequate visualization of the mitochondrial network. The mitochondrial network fluorescence and volume was identified and analyzed using the Volocity software (PerkinElmer, Waltham, MA). The mitochondria in cortex were set as 100% in mice control sections enabling a comparison between different groups.

### RNA extraction and PCR

Total RNA was isolated using Tripure reagent (Roche Diagnostics, Basel, Switzerland) and then reverse-transcribed into cDNA using the Quantitect Enzyme Reverse Transcriptase Kit (Qiagen) according to the manufacturer’s instructions. SYBR Green real-time RT-PCR was performed with the Stratagene Mx3005P sequence detection system (Agilent Technologies) using SensiMix SYBR Low-ROX (Bioline, Luckenwalde, Germany). The primers used in the RT-PCR to determine the expression levels of TNF, IL1β, IL6, Socs3, Stat3, NPY, Actβ, and Rn18s were predesigned and validated by Qiagen (QuantiTect Primer Assay) and are presented in Additional file 1: Table S1. Threshold cycle values were determined automatically using the software supplied by Stratagene (MxPro Software 3.20). Primer specificity was verified by melt curve analysis. Relative gene expression was determined using the 2^ΔΔC^_T_ method. All values for gene expression following PCR analysis are expressed as relative to reference genes content and referred to as relative expression.

### Protein analyses

Cortical homogenates containing 20 μg of protein were loaded on a 4–20% SDS-PAGE gel (BioRad) and transferred onto PVDF membranes (Millipore). They were then incubated with an antibody cocktail targeting one representative subunit of each of the five mitochondrial complexes (MitoSciences, complex I subunit NDUFB8, complex II-30 Da, complex III Core protein 2, complex IV subunit I, complex V alpha subunit), and mouse anti-mitofusin2 (Mfn2) (Abcam). Immunoreactive protein was detected using an enhanced chemiluminescence reagent (ECL Clarity, Bio Rad). Densitometric quantification was carried out with ImageQuant TL Image Analysis software version 7.0 (LAS 4000, GE Healthcare). Protein loading was monitored using a mouse monoclonal antibody against β-actin (Sigma-Aldrich).

### Data analysis

Statistical analysis data are presented as mean ± SEM. Post hoc comparisons between two groups were done with Student’s *t* test. One-way ANOVA, followed by a Tukey’s post hoc test, was used for comparing multiple groups. All calculations were made using SPSS v15.0 software (Chicago, IL, USA). Statistical significance was set at *P* < 0.05.

Detailed protocols are described in Supplemental Experimental Procedures.

## Results

### Endothelial-specific megalin deficiency leads to obesity

**Fig. 1:**
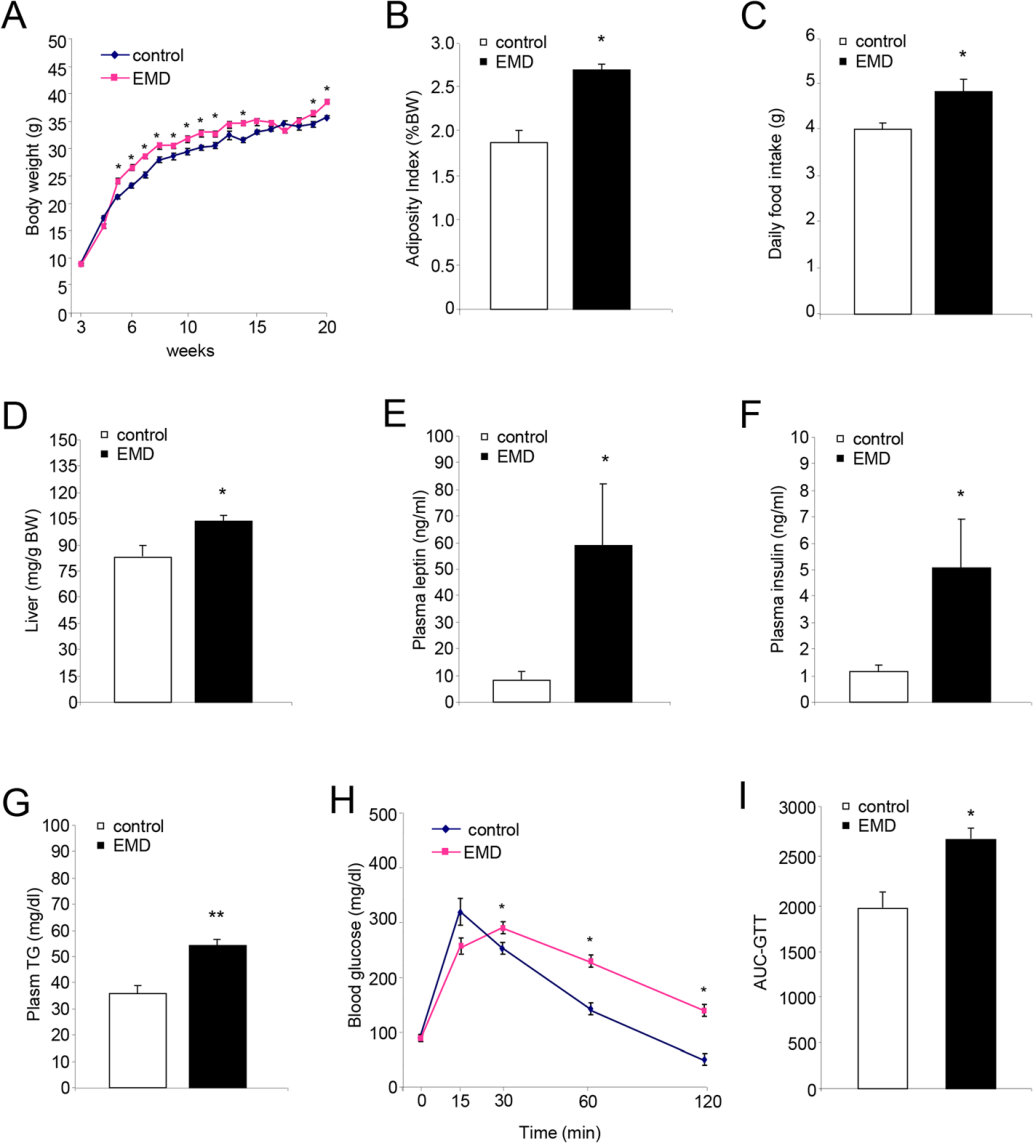
Analysis of energy homeostatic and metabolic parameters. EMD (*n* = 12) and control (*n* = 10) mice were evaluated for 20 weeks. **a** Weekly body weight, measured along 20 weeks, was significantly increased in EMD mice compared to wt mice. **b** Adiposity index was significantly increased in EMD mice compared to wt mice. **c** Daily food intake was increased in EMD mice compared to wt mice. **d** Relative weight of liver was reduced in EMD mice compared to wt mice. (**e–g**) **e** Plasma levels of leptin, **f** insulin, and **g** triglycerides were significantly increased in EMD mice compared to wt mice. **h** Glucose tolerance was assessed over 120 min in EMD mice compared to wt mice. **i** The area under curve (AUC) was calculated for respective groups and used for statistical analysis. *TG* triglycerides. Data are presented as mean ± SEM. *P < 0.05, **P < 0.01; Student’s *t* test.

To investigate the impact of megalin knockdown in BBB on energy balance homeostasis, body weight of EMD and littermate control mice was monitored from weaning until 20 weeks of age. Megalin deletion led to increased body weight at 5 weeks (*P* < 0.05) that persisted over time (Fig. 1a). In agreement with this, EMD mice showed increased body adiposity index (BAI), defined as the percentage of fat in the body (control = 1.8 ± 0.2%; EMD = 2.7 ± 0.1%; *P* < 0.05; Fig. 1b). Along this observation, EMD mice exhibited increased cumulative food intake (control = 4.0 ± 0.1 g/day; EMD = 4.8 ± 0.4 g/day; *P* < 0.05; Fig. 1c) and significantly higher relative weight (weight ratio to body weight) of the liver control = 80.6 ± 5.8 mg/g BW; EMD = 102.1 ± 3.3 mg/g BW *P* < 0.05; Fig. 1d). Moreover, EMD mice displayed increased plasma levels of leptin, (control = 7.5 ± 4.3 ng/ml; EMD = 59.7 ± 27.5 ng/ml; *P* < 0.05; Fig. 1e), insulin (control = 1.7 ± 0.7 ng/ml ; EMD = 5.2 ± 1.6 ng/ml; *P* < 0.05; Fig. 1f), and triglycerides (control = 35.2 ± 4.9 mg/dl; EMD = 52.4 ± 3.8 mg/dl; *P* < 0.01; Fig. 1g) compared to control mice. These phenotypes were concomitant with reduced glucose tolerance in the EMD compared to control mice as evaluated in a glucose tolerance test (Fig. 1h, i). Together, these results indicate that deletion of the transporter megalin in brain endothelial cells leads to mild obesity in mice.

### Leptin signaling is disrupted in the hypothalamus of EMD mice

**Fig. 2:**
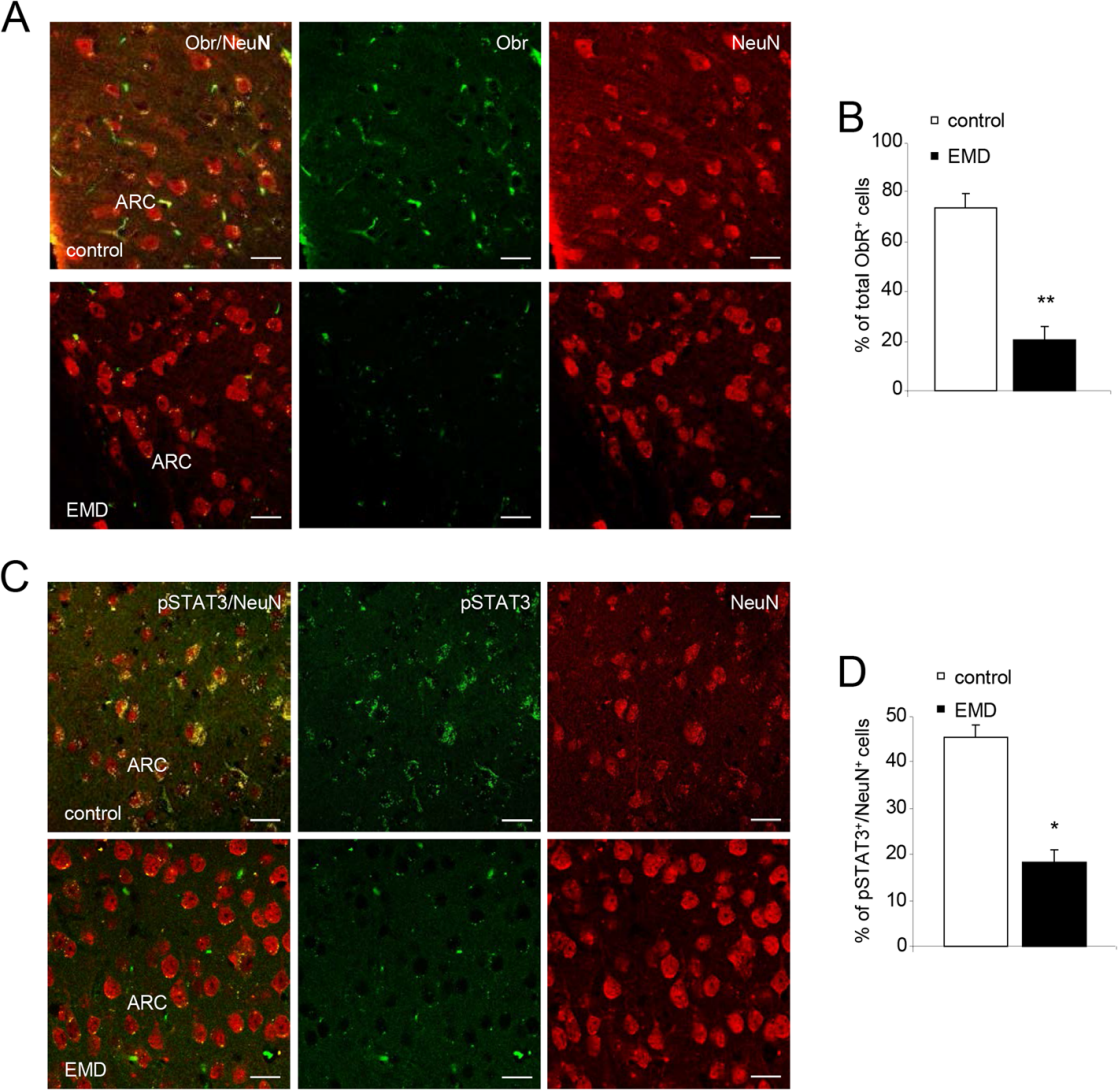
Analysis of Obr and pSTAT3 expression in the hypothalamus. **a** Confocal images showing Obr (*green*) in neurons immunostained with anti-NeuN (*red*) staining in EMD and control mice. Scalebar = 20 μm. **b** Percent of Obr^+^ cells that were NeuN^+^ diminished in EMD mice compared to control mice. **c** Confocal images showing pSTAT3 (*green*), and NeuN (*red*) staining in EMD and control mice. Scalebar = 20 μm. **d** Quantitation of pSTAT3-positive NeuN neurons in hypothalamic sections of EMD and control mice. Data are presented as mean ± SEM. *ARC* arcuate nucleus. *P < 0.05, **P < 0.01; Student’s *t* test.

The impact of leptin signaling on hypothalamus after BBB megalin deletion was evaluated. It is known that leptin binds to ObR leading to the phosphorylation of STAT3 [11–13]. Reduced ObR immunoreactivity was observed in hypothalamic neurons from 20-week-old EMD mice compared to controls (control = 73.8 ± 6.1%; EMD = 19.3 ± 3.8%; *P* < 0.01; Fig. 2a, b). Levels of pSTAT3 were also reduced in hypothalamic neurons of EMD compared to control mice (control = 45.1 ± 3.2%; EMD = 18.9 ± 2.8%; *P* < 0.05; Fig. 2c, d). These results suggest that the mild obese phenotype of EMD mice could be a consequence of hypothalamic leptin resistance. The ObR and pSTAT3 downregulation showed above reflects leptin resistance in the EMD mice.

### Megalin knock down in the BBB induces cortical and hippocampal inflammation

**Fig. 3:**
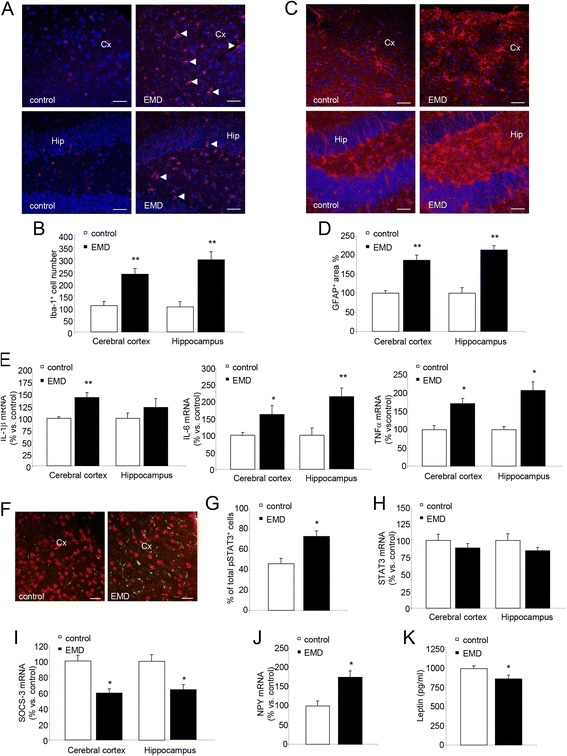
EMD mice showed exacerbated cortical and hippocampal inflammation. **a** Higher expression of Iba-1 was observed in both cerebral cortex and hippocampus in EMD mice compared to control mice. Scalebar = 20 μm. **b** Quantitative analysis of the number of Iba-1 stained cells was performed in both brain areas. **c** Higher expression of GFAP was observed in both cerebral cortex and hippocampus in EMD mice compared to control mice. Scalebar = 20 μm. **d** Quantitative analysis of the percentage area covered by GFAP immunoreactivity was performed in both brain areas. Data are given as mean ± SEM. **e** mRNA cytokine expression was analyzed. Higher mRNA expression of interleukin (Il)-1β, Il-6, and tumor necrosis factor (TNF-α) were observed in cerebral cortex and hippocampus in EMD mice compared to control mice. Neurogenesis impairment due to increased inflammation is reflected in Additional file 1: Fig. S1. **f** Confocal images showing pSTAT3 (*green*) and NeuN (*red*) staining in EMD and control mice. Scalebar = 20 μm. **g** Quantization of pSTAT3^+^ neurons was performed in cerebral cortex in EMD and control mice. **h** Unchanged mRNA STAT3 expression was observed in both cerebral cortex and hippocampus in EMD mice compared to control mice. **i** mRNA SOCS3 expression was analyzed in both cerebral cortex and hippocampus in EMD mice compared to control mice. Diminished mRNA expression of SOCS3 was observed in both brain areas. **j** Higher mRNA expression of NPY was found in cerebral cortex from EMD mice compared to control mice. **k** Protein levels of leptin were lower in EMD compared to control mice. Data are given as mean ± SEM. *Cx* cerebral cortex, *Hip* hippocampus. Data are presented as mean ± SEM. *P < 0.05, **P < 0.01; Student’s *t* test.

The analysis of Iba-1 and GFAP fluorescence in cortical and hippocampal sections reflected significantly higher glial activation in the EMD mice when compared to the control mice (Iba-1 fluorescence: in cortex, control = 100.0 ± 23.0%; EMD = 212.2 ± 28.4%; *P* < 0.01; in hippocampus, control = 100.0 ± 25.2%; EMD = 309.4 ± 42.1%; *P* < 0.01; Fig. 3a, b; GFAP fluorescence: in cortex, control = 100.0 ± 15.8%; EMD = 193.5 ± 35.4%; *P* < 0.01; in hippocampus, control = 100.0 ± 18.4%; EMD = 219.9 ± 22.4%; *P* < 0.01; Fig. 3c, d). Similar results were found in hypothalamic sections showing increased glial activation (Iba-1 fluorescence in hypothalamus: control = 100 ± 10%; EMD = 145 ± 10%; *P* < 0.002, Additional file 1: Fig. S1 a, b; GFAP fluorescence in hypothalamus: control = 100 ± 5%; EMD = 171 ± 27%; *P* < 0.005, Additional file 1: Figs. S1 c, d). A number of events related to pro-inflammatory cytokines secretion can be observed after microglial and astroglial activation in brain. The mRNA expression analysis of pro-inflammatory cytokines showed increased mRNA levels in the EMD mice group compared to control mice of interleukin 1β (IL-1β) (41.4%; *P* < 0.05 in cerebral cortex), interleukin-6 (IL-6) (62.5%; *P* < 0.01 in cerebral cortex; and 113.9%; *P* < 0.05 in hippocampus) and tumor necrosis factor-α (TNF-α) (72.7%; *P* < 0.05 in cerebral cortex; and 102.2%; *P* < 0.05 in hippocampus) (Fig. 3e). It is known that these pro-inflammatory cytokines activate the STAT3 transcription factor. Densitometric analysis of pSTAT3 signal in cortical sections showed increased phosphorylation levels in EMD mice compared to wt (Figs. 3f, g) while the STAT3 expression levels remained unchanged (Fig. 3h). Additionally, the mRNA expression of the suppressor of cytokine signaling-3 (SOCS3) was found significantly reduced in the cortical and hippocampal sections from the EMD mice compared to those sections from the control group (39.72%; *P* < 0.05 in cerebral cortex; and 38.02%; *P* < 0.05 in hippocampus; Fig. 3i). Moreover, the mRNA expression of the orexigenic neuropeptide Y (NPY) which is involved in the energetic balance regulation along leptin, was found significantly increased in the EMD mice cortical samples compared to the control mice (69.6%; *P* < 0.050 (Fig. 3j). Accordingly, the leptin levels in cortex from the the transgenic mouse model were found reduced compared to control (control = 1037.1 ± 74.1 pg/ml; EMD = 906.5 ± 25.0 pg/ml; *p* < 0.05; Fig. 3k). The analysis of the pSTAT3 signal, NPY expression, and leptin levels in the hippocampus reported no significant changes (Additional file 1: Fig. S2).

Additionally, increased glial activation affects the neurogenesis. Indeed, the analysis of doublecortin (DCX)-positive immature neurons and the polysialic acid-neural cell adhesion molecule (PSA-NCAM)-positive mature neurons in the dentate gyrus of the hippocampus, revealed that neurogenesis is impaired in the EMD mice compared to control (DCX^+^: control = 35.4 ± 4.6 cells, EMD = 14.0 ± 2.7 cells, *P* < 0.01, Additional file 1: Fig. S3 a, b; PSA-NCAM^+^: control = 25.6 ± 2.8 cells; EMD = 6.0 ± 1.7 cells; *P* < 0.05; Additional file 1: Figs. S3c, d).

### BBB megalin deletion is related to mitochondrial impairment

**Fig. 4:**
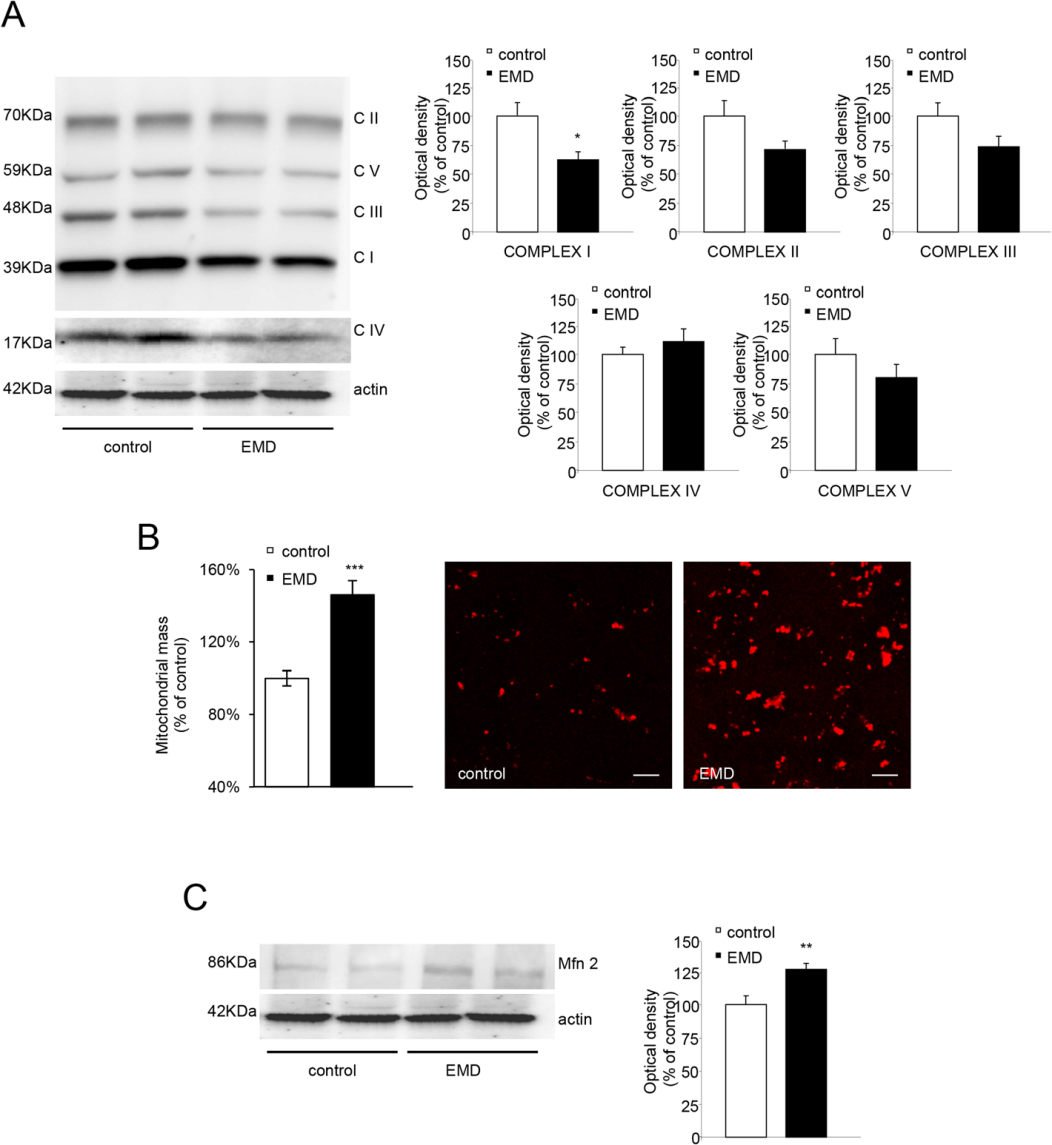
Mitochondrial impairments in the EMD mice cerebral cortex. **a** OXPHOS subunits in EMD and control mice. Representative OXPHOS bands of cortical samples are shown (*left panels*). Protein levels of representative subunits of each of the five OXPHOS complexes were quantified (*right panels*). **b** Mitochondrial mass assessment performed in different regions from cortical sections from all EMD and control mice. Quantitative analysis of fluorescence targeting the ATP synthase β subunit revealed higher mitochondrial mass in EMD mice compared to control mice (*left panels*). **c** Representative western blot is shown for mitofusin 2 (Mfn2). *Right panel* shows increased protein levels of Mfn2 in EMD mice compared to control mice. Data are presented as mean ± SEM. *P < 0.05; Student’s *t* test.

Obesity has been linked to mitochondrial alterations, [14, 15]. To evaluate possible mitochondrial impairments in our experimental model, the protein levels of representative subunits from the ETC and OXPHOS complexes were analyzed by immunoblotting in cortical samples from the wt and the EMD mice. A significant reduction in the complex I component was found in the EMD mice cortical tissue compared to those from the wt (Fig. 4a). Downregulated complexes could indicate energy metabolism disturbances and cells may compensate this deficit by mitochondrial biogenesis stimulation [16]. Indeed, the analysis of cortical sections in the EMD mice revealed increased mitochondrial mass compared to the control (control = 100 ± 4%; EMD = 146 ± 8%; n = 36; *P* < 0.0001; Fig. 4b). This observation, along with the increased mitofusin 2 (Mfn2) levels found in samples from cortical tissue in the EMD, suggests a compensatory mechanism for the deficient EMD mitochondria (Fig. 4c).

## Discussion

In the present study, the potential role of megalin in the energy homeostasis maintenance was investigated. Even though it is expressed in several tissues, brain endothelium is the only one endothelial tissue where megalin is expressed simultaneously with Tie2. That situation makes the Cre recombinase megalin deletion system specific to brain endothelium. We demonstrate that the silencing of megalin in the mouse brain endothelium is sufficient to increase the food intake, weight gain, and adiposity triggering hyperleptinemia, hyperinsulinemia, increased triglycerides blood levels, and impaired glucose tolerance. Additionally, we provide evidences supporting the physiological role of megalin in the regulation of energy balance in agreement with previous data [15–17].

We reported here the first evidence that megalin blockage in BBB endothelial cells reduce the brain leptin uptake leading to hyperphagia and obesity. A recent review summarizes a number of data showing that “Western diets” enriched of saturated fatty acids and simple carbohydrates contribute to degrade the BBB integrity by reducing the expression of tight junction proteins, particularly claudin 5 and 12 [18]. This BBB dysfunction and disruption may be followed by leukocyte infiltration into the brain causing or aggravating neuroinflammation. Thus, in our experimental model we cannot exclude a disruption in the integrity of the BBB, as EMD mice share the phenotype with other obesity mice models showing increased levels of leptin, insulin and triglycerides in plasma, and the contribution/side effects of inflammatory cell infiltration from blood into the brain. Our findings account for the megalin expression in cerebral microvessel endothelial cells which is capable to bind and endocyte leptin. Moreover, our results are in agreement with a recent study suggesting megalin as the primary leptin transporter at the BBB instead of ObR [19]. In this work, the authors still detected leptin transport across monolayers of cultured brain endothelial cells in the presence of a ObR-neutralizing antibody [19]. Our previous findings showed that blocking megalin expression in the choroid plexus cells resulted in lower leptin levels in the cerebrospinal fluid caused by impairments in the leptin transport [3]. Significantly high serum leptin levels were presented in obese subjects compared to a healthy control population suggesting the leptin resistance as the main risk factor to induce obesity [20, 21]. Studies showed that endothelial specific leptin receptor knockout elicits persistent leptin transport even increasing the leptin uptake into the brain parenchyma [22, 23]. These findings could suggest an additional leptin brain gate/transporter mediated by an alternative receptor/endocytic transporter other than ObR. Our results suggest that blood leptin handle megalin to cross the BBB reaching the neuronal receptors and triggering the leptin signaling cascade as supports the reduced STAT3 activation in the hypothalamic neurons from the EMD mice. We also found reduced neuronal ObR expression in the EMD mice possibly as blood hyperleptinemia consequence in agreement with previous studies [24].

The relationship between obesity-induced inflammation and brain plasticity, particularly in the hippocampus and frontal cortex, is highlighted by the enhanced astrocytic and microglial activation and increased mRNA levels of IL-1β, IL-6, and TNF-α in the cortex and the hippocampus of EMD mice compared to the control. The obesity-related glial upregulation has been found also in brain regions involved in cognition and memory from patients with clinical risk factors for stroke and obesity mouse models [25, 26]. Neuroinflammation can be induced by both CNS-intrinsic factors and systemic influences (factors from outside the CNS), resulting in peripheral overproduction of pro-inflammatory cytokines, such as IL-1β, IL-6, and TNF-. These circulatory inflammatory mediators cross the BBB and may lead to CNS inflammation, blocking intracellular signaling of molecules such as insulin or leptin. Conversely, the activation of glial cells results in a constant production of inflammatory cytokines and chemokines by these cells, resulting in a vicious circle affecting surrounding CNS cells, including astrocytes and neurons, and finally causing neurodegeneration [27]. Additionally, the EMD mice showed increased activation of STAT3. The activation of the JAK/STAT3 signaling pathway could be a downstream effect of the CNS inflammation as occurs in neurodegenerative disorders [28]. Altered IL-1β, IL-6, and TNF-α expression levels activating the STAT3 pathway have been found in brain inflammation models [29, 30]. Another upstream player in the JAK/STAT3 pathway is the suppressor SOCS3 which avoids the STAT3 phosphorylation [31]. Our data show reduced SOCS3 mRNA expression in the EMD mice. SOCS3 blocks the upregulated expression of inflammatory mediators, including IL-6, and it is downregulated in chronic inflammatory diseases [32, 33]. Our results could indicate SOCS3-mediated pSTAT3 signaling may be responsible to the continued production of cytokines and the subsequent neuroinflammation found in the EMD mice. Along with the reduced SOCS3, we found increased NPY expression in the EMD mice, indicating impaired leptin signaling pathway as it demonstrates the reduced leptin levels. It is well established that leptin induces SOCS3 expression and inhibits the NPY [34]. NPY plays a role in a variety of basic physiological functions and is also implicated regulating cognition, including anxiety, learning, and memory [35]. We previously demonstrated increased anxiety behavior and impaired learning ability and recognition in the memory in the EMD mice [10] that could be related to the high NPY expression now demonstrated in the cerebral cortex. Neuroinflammation has been identified as a primary assaulting candidate for impaired neurogenesis [36]. We propose the excessive release of inflammatory cytokines such as Il-1, Il-6, and TNF-α, may be downregulating cell survival and differentiation, and subsequently, neurogenesis. This has been further documented by other studies where hippocampal and hypothalamic neurogenesis was restored after blocking inflammatory signaling [37, 38].

A significant reduction in the complex I protein levels were found in the EMD mice, suggesting possible energetic disturbances. Modulating the mitochondrial quality and function, leptin may control the cell bioenergetic efficiency [39]. We can speculate that impaired leptin signaling in the EMD mice may be responsible for mitochondrial disturbances suggesting a link between obesity and brain mitochondrial dysfunction, and further, mice learning impairments [38–41]. Neuroinflammation associated with neurodegenerative diseases, like AD, is also linked to mitochondrial impairment [39–41]. Thus, EMD mitochondrial defects in EMD mice could be related to central inflammation observed in these mice. Along with the reduced complex I protein levels in the EMD mice, increased mitochondrial mass were found in the EMD mice brains compared to the control. The maintenance of mitochondrial homeostasis is central to keep the neuronal viability and function. The higher mitochondrial mass observed in the EMD mice could compensate the energy depletion as a consequence of complex I failure. Similar observations were found in muscle tissue from rats where a reduction in OXPHOS efficiency induced enhanced mitochondrial biogenesis as mitochondrial adaptation [42]. We additionally found increased Mfn2 levels in brains from the EMD mice. Increased Mfn2 levels could indicate enhanced mitochondrial biogenesis as has been previously demonstrated Mfn2 regulates this process. [43, 44].

## Conclusions

Our findings demonstrate that the EMD mice constitute a valuable obesity model, linking obesity and neurodegeneration. Here, we demonstrate deletion of megalin in brain endothelial cells to be a novel mechanism to promote obesity and to activate obesity-induced neuropathological mechanisms.

## Additional file

Additional file 1: Figure S1. EMD mice showed exacerbated hypothalamic inflammation. (A) Higher expression of Iba-1 was observed in arcuate hypothalamic nucleus in EMD mice compared to control mice. **(**B) Quantitative analysis of the percentage area covered by Iba-1 immunoreactivity was performed in this brain area. (C) Higher expression of GFAP was observed in arcuate hypothalamic nucleus in EMD mice compared to control mice. (D) Quantitative analysis of the percentage area covered by GFAP immunoreactivity was performed in this brain area. *Arc* arcuate hypothalamic nucleus. Data are presented as mean ± SEM. ** *P <* 0.01; Student’s *t* test. **Figure S2**. Hippocampal NPY and leptin expression in EMD mice. (A) mRNA expression of NPY was unchanged in hippocampal samples from EMD mice compared to control mice. (B) Protein levels of leptin were unaffected in EMD compared to control mice. Data are given as mean ± SEM. **Figure S3**. Neurogenesis is reduced in EMD mice. (A) Representative photomicrographs of DCX (*red*) and DAPI nuclei (*blue*) staining in the hippocampal granule cell layer of EMD (*n* = 8), and control (*n* = 7) mice. Scalebar = 20 μm. (B) Number of DCX+ cells in the DG of hippocampus is reduced in EMD mice compared to control mice. (C) Fluorescent immunostaining for PSA-NCAM mainly localized in the middle and inner cell layers of the dentate gyrus in EMD (*n* = 8) and control (*n* = 7) mice. Scalebar = 20 μm. (D) PSA-NCAM+ cell number is reduced in the DG of hippocampus in EMD mice compared to control mice. Data are presented as mean ± SEM. *DG* dentate gyrus. **P <* 0.05, ***P <* 0.01; Student’s *t* test. Related to Fig. 3. **Table S1**. primers used for RT-PCR. Related to Fig. 3. (DOCX 844 kb)
