# Supplementary material for: Obesity and neuroinflammatory phenotype in mice lacking endothelial megalin
Source: J Neuroinflammation. 2017 Jan 31;14:26. doi: 10.1186/s12974-017-0800-2 (PMC5282716; doi:10.1186/s12974-017-0800-2)
Supplement: Additional file 1: Figure S1. — EMD mice showed exacerbated hypothalamic inflammation. (A) Higher expression of Iba-1 was observed in arcuate hypothalamic nucleus in EMD mice compared to control mice. (B) Quantitative analysis of the percentage area covered by Iba-1 immunoreactivity was performed in this brain area. (C) Higher expression of GFAP was observed in arcuate hypothalamic nucleus in EMD mice compared to control mice. (D) Quantitative analysis of the percentage area covered by GFAP immunoreactivity was performed in this brain area. Arc arcuate hypothalamic nucleus. Data are presented as mean ± SEM. ** P < 0.01; Student’s t test. Figure S2. Hippocampal NPY and leptin expression in EMD mice. (A) mRNA expression of NPY was unchanged in hippocampal samples from EMD mice compared to control mice. (B) Protein levels of leptin were unaffected in EMD compared to control mice. Data are given as mean ± SEM. Figure S3. Neurogenesis is reduced in EMD mice. (A) Representative photomicrographs of DCX (red) and DAPI nuclei (blue) staining in the hippocampal granule cell layer of EMD (n = 8), and control (n = 7) mice. Scalebar = 20 μm. (B) Number of DCX+ cells in the DG of hippocampus is reduced in EMD mice compared to control mice. (C) Fluorescent immunostaining for PSA-NCAM mainly localized in the middle and inner cell layers of the dentate gyrus in EMD (n = 8) and control (n = 7) mice. Scalebar = 20 μm. (D) PSA-NCAM+ cell number is reduced in the DG of hippocampus in EMD mice compared to control mice. Data are presented as mean ± SEM. DG dentate gyrus. *P < 0.05, **P < 0.01; Student’s t test. Related to Fig. 3. Table S1. primers used for RT-PCR. Related to Fig. 3. (DOCX 844 kb) [file 12974_2017_800_MOESM1_ESM.docx]

**Additional file 1**

**
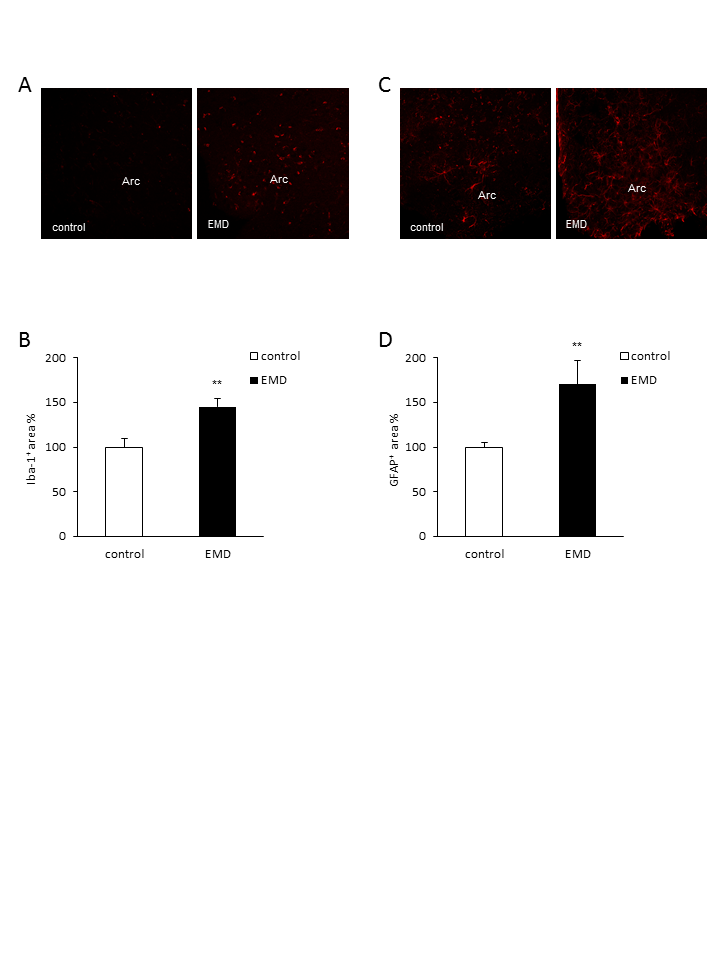
**

**Figure S1**. **EMD mice showed exacerbated hypothalamic inflammation.** (A) Higher expression of Iba-1 was observed in arcuate hypothalamic nucleus in EMD mice compared to control mice. (B) Quantitative analysis of the percentage area covered by Iba-1 immunoreactivity was performed in this brain area. (C) Higher expression of GFAP was observed in arcuate hypothalamic nucleus in EMD mice compared to control mice. (D) Quantitative analysis of the percentage area covered by GFAP immunoreactivity was performed in this brain area. Arc, arcuate hypothalamic nucleus. Data are presented as mean ± SEM. ** *P* < 0.01; Student’s *t*-test.

**
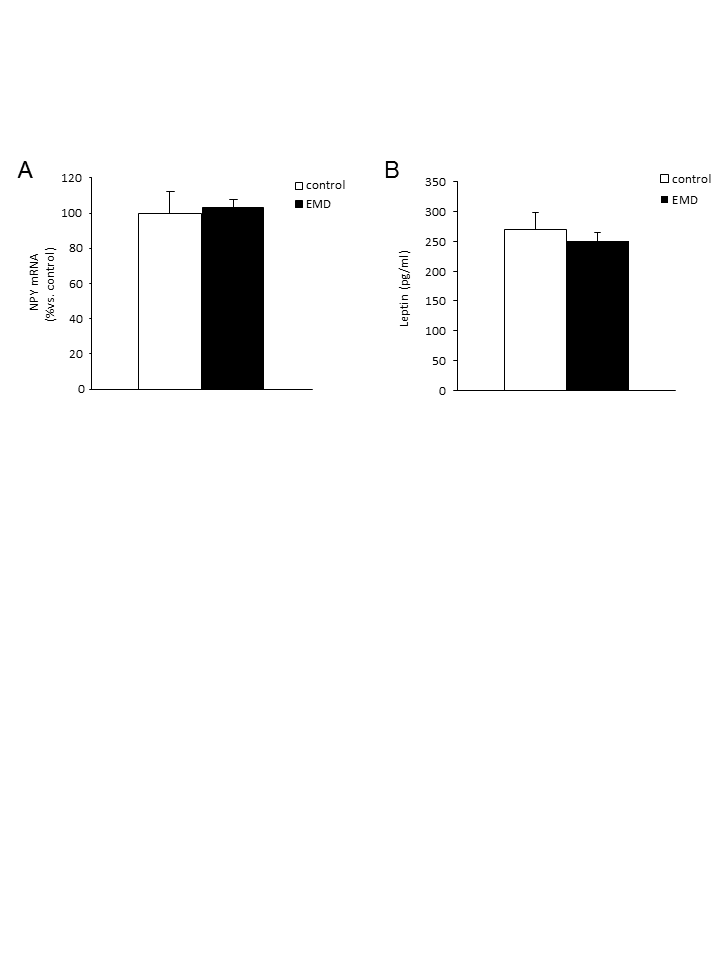
**

**Figure S2**. **Hippocampal NPY and leptin expression in EMD mice.** (A) mRNA expression of NPY was unchanged in hippocampal samples from EMD mice compared to control mice. (B) Protein levels of leptin were unaffected in EMD compared to control mice. Data are given as mean ± SEM.

**
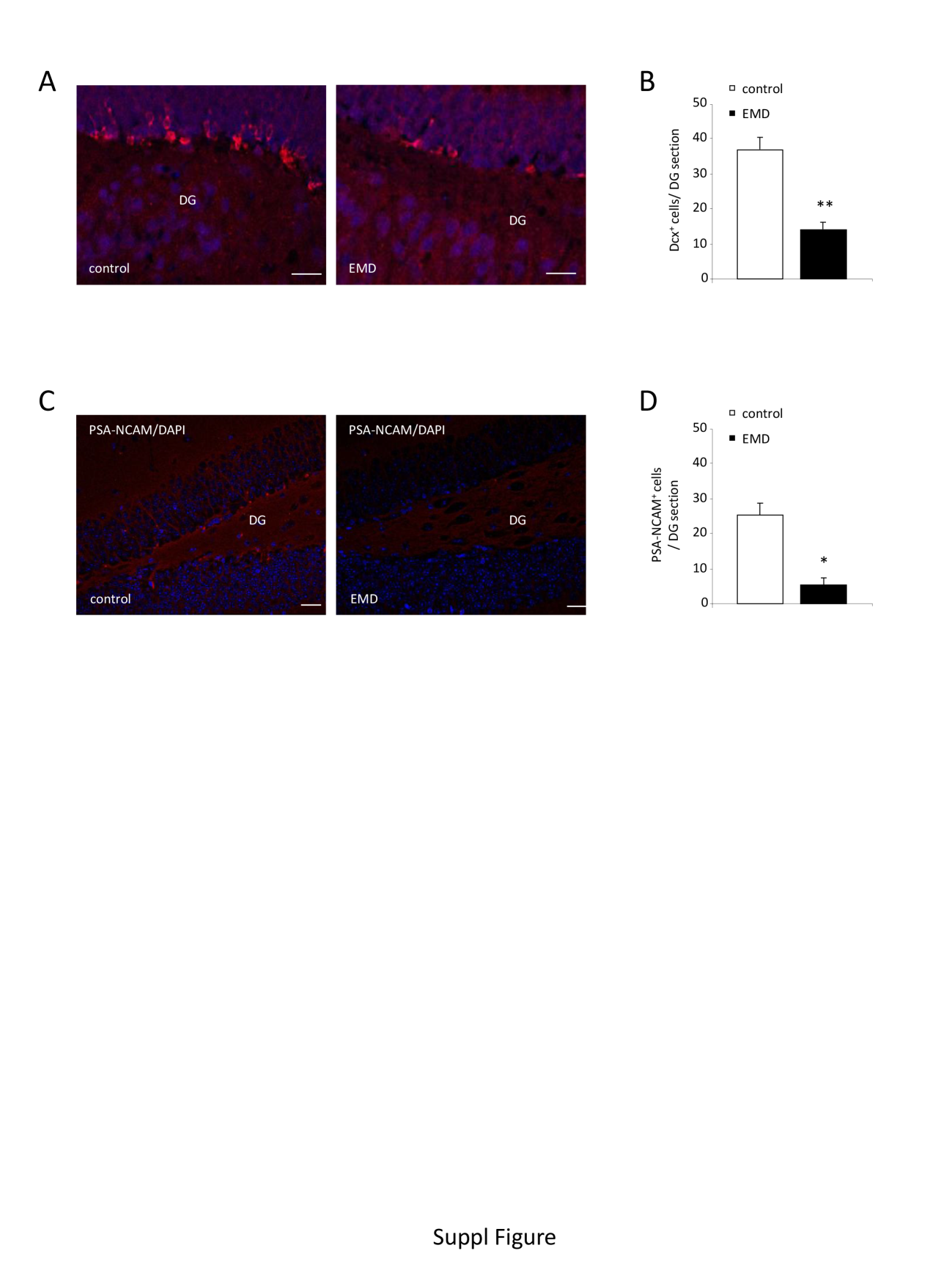
Figure S3. Neurogenesis is reduced in EMD mice.** (A) Representative photomicrographs of DCX (red), and DAPI nuclei (blue) staining in the hippocampal granule cell layer of EMD (n= 8), and control (n= 7) mice. Scale bar = 20 μm. (B) Number of DCX^+^ cells in the DG of hippocampus is reduced in EMD mice compared to control mice. (C) Fluorescent immunostaining for PSA-NCAM mainly localized in the middle and inner cell layer of the dentate gyrus in EMD (n= 8), and control (n= 7) mice. Scale bar = 20 μm. (D) PSA-NCAM^+^ cell number is reduced in the DG of hippocampus in EMD mice compared to control mice. Data are presented as mean ± SEM. DG, dentate gyrus. **P*<0.05, ** *P* <0.01; Student’s *t*-test. Related to **Figure 3**.

**Table S1:** Primers used for RT-PCR. Related to **Figure 3**.

| **Gene symbol**  **(mouse)** | **Name** | **Cat.No.** | **Amplicon**  **Length (bp)** | **Gene Bank**  **Accession**  **Number** |
| --- | --- | --- | --- | --- |
| **TNF** | Tumor necrosis factor | QT00104006 | 112 | NM_013693 |
| **IL1β** | Interleukin 1beta | QT01048355 | 150 | NM_008361 |
| **IL6** | Interleukin 6 | QT00098875 | 128 | NM_031168 |
| **Socs3** | Suppressor of cytokine signaling 3 | QT02488990 | 90 | NM_007707 |
| **Stat3** | Signal transducer and activator of transcription 3 | QT00148750 | 99 | NM_213659 |
| **Npy** | Neuropeptide Y | QT00134302 | 150 | NM_023456 |
| **Actβ** | Actin, beta | QT01136772 | 77 | NM_007393 |
| **Rn18s** | 18S ribosomal RNA | PPM72041A-200 | 121 | [NR_003278](http://www.ncbi.nlm.nih.gov/entrez/query.fcgi?holding=&db=Nucleotide&cmd=search&term=NR_003278) |

**Supplemental Experimental Procedures**

***EMD mouse phenotype study***

EMD and **control** mice were grouped under a 12 h light: 12 h dark schedule and allowed *ad libitum* access to food and water.

Body weight was monitored weekly throughout the study. Daily food intake was calculated as the average intake of chow. At the end of experiments, animals were anaesthetised with isoforane, blood was drawn, and perfused transcardially with saline buffer or 4% paraformaldehyde in 0.1 M phosphate buffer (PB, pH 7.4) for biochemical and immunohistochemical analysis, respectively. Then, brain, liver, and adipose tissue, were collected for further processing and stored at -80°C until analysis. The liver and adipose tissue were previously weighed. Glucose tolerance test was assessed prior to sacrifice.

***Glucose tolerance test (GTT)***

Glucose tolerance test was performed in 16-h-fasted animals. After collection of a baseline sample, mice received an intraperitoneal (i.p.) injection of glucose (2 g/kg body weight). Blood glucose levels were detected after 15, 30, 60 and 120 min after glucose injection, using a Compact Plus Glucose Meter (Accu-Chek, Roche). Area under the curve (AUC) was used to compare glucose tolerance between groups.

***Immunohistochemistry***

For immunohistochemistry assays, fixed brains were cut on a vibratome (Leica Microsystems) at 30 µm, and tissue sections were collected in cold 0.1 M PB, After overnight incubation, primary antibody staining was revealed using fluorescence-conjugated secondary antibodies from Molecular Probes. Images were obtained by confocal microscopy.

Fluorescent images were obtained by laser confocal microscopy (Carl Zeiss-LSM510) using the excitation lasers 405, 488 and 568 for DAPI, green and red fluorescence, respectively. For quantitative analysis of hypothalamic Obr^+^/NeuN^+^ neurons, and Obr^+^/GFAP^+^ astrocytes, double fluorescence cells were counted, marked to prevent multiple counts, and expressed as percentage of total GFAP^+^ or NeuN^+^ double labelled cells. For quantitative analysis of hypothalamic pSTAT3^+^/NeuN^+^ neurons, double fluorescence cells were counted, marked to prevent multiple counts, and expressed as percentage of total NeuN^+^ neurons. The number of reactive microglial Iba1^+^ cells was estimated by unbiased stereology method as previously described (Anitua et al., 2015). GFAP^+^ area was stereologically analyzed as described previously (Perez-Gonzalez et al., 2014). In addition, one series of sections was used for double-labeling experiments using DAPI nuclear staining (Sigma), and DCX as a marker of neuronal progenitor cells. Using the Cell Counter program of the NIH ImageJ software, we counted hippocampal DCX^+^ cells of the captured confocal microphotographs.

***Primers***

The primers targeting the mouse genes (IL-1β: Mm_ILβ_2_SG; IL-6: Mm_IL_6_1SG; TNFα: Mm_Tnf_1_SG; SOCS3: Mm_SOCS3_1_SG, STAT3: Mm_Stat3_1_SG and NPY: Mm_Npy_1_SG) and the reference genes beta actin (Mm_Actb_2_SG) and 18S (Mm_Rn18s_3_SG) were predesigned and validated by Qiagen (QuantiTect Primer Assay). Primers pairs used are shown in Supplementary Table. Threshold cycle values were determined automatically using the software supplied by Stratagene (MxPro Software 3.20). Primer specificity was verified by melt curve analysis. Relative gene expression was determined using the 2^ΔΔC^_T_ method. All values for gene expression following PCR analysis are expressed as relative to reference genes content and referred to as relative expression.

**References**

Anitua, E., Pascual, C., Perez-Gonzalez, R., Orive, G., and Carro, E. (2015). Intranasal PRGF-Endoret enhances neuronal survival and attenuates NF-kappaB-dependent inflammation process in a mouse model of Parkinson's disease. J Control Release *203*, 170-180.

Perez-Gonzalez, R., Alvira-Botero, M.X., Robayo, O., Antequera, D., Garzon, M., Martin-Moreno, A.M., Brera, B., de Ceballos, M.L., and Carro, E. (2014). Leptin gene therapy attenuates neuronal damages evoked by amyloid-beta and rescues memory deficits in APP/PS1 mice. Gene Ther *21*, 298-308.
